# Calcification in Thoracic Splenosis

**DOI:** 10.1155/2022/9538355

**Published:** 2022-10-11

**Authors:** Tofura Ullah, Sneh Chauhan, Joseph Friedman, Gideon Yoeli, Maximo Mora, Craig A. Thurm

**Affiliations:** ^1^Department of Clinical Research, MediSys Health Network, Jamaica, NY 11418, USA; ^2^Department of Pulmonary Medicine, Jamaica Hospital Medical Center, Jamaica, NY 11418, USA; ^3^Department of Radiology, Jamaica Hospital Medical Center, Jamaica, NY 11418, USA; ^4^Department of Pathology, Jamaica Hospital Medical Center, Jamaica, NY 11418, USA

## Abstract

Splenosis is a rare condition described as the implantation of ectopic splenic tissue, usually after a splenic rupture. Thoracic splenosis refers to acquired ectopic splenic tissue found within the thoracic cavity, often caused by thoracoabdominal trauma or surgery. Most cases are asymptomatic and many years may elapse before they are incidentally discovered on chest radiography or thoracic computed tomography. Splenosis is often misinterpreted as a malignancy on initial imaging. We wish to highlight a rare case of thoracic splenosis presenting with calcified and non-calcified nodules. Only two other cases of calcification have been reported in intrathoracic splenosis, neither of which provided CT images of this finding.

## 1. Introduction

Splenosis describes ectopic splenic tissue found in patients after rupture of the spleen. Splenic fragments are often found within the abdominal and pelvic cavities. Less commonly, these splenic implants can also be discovered in the thoracic cavity. This occurs in approximately 18% of patients after splenic insult [1]. Most cases of thoracic splenosis are diagnosed several years after thoracoabdominal trauma or surgery. In a review of thirty-eight cases, the average reported time interval between abdominal trauma requiring splenectomy and detection of thoracic splenosis has been reported to be 21 years, with a range of 3-45 years [2]. Here, we report a case of thoracosplenosis that was diagnosed 26 years after a gunshot wound. We would like to highlight the presence of calcification within these lesions, which appears to be rare. Recognizing that calcification can be seen in this entity may avoid diagnostic errors when evaluating such lesions.

## 2. Case Presentation

**Figure 1:**
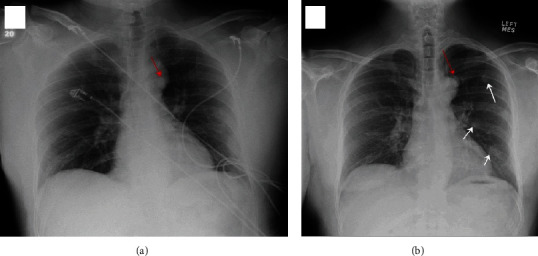
Chest X-ray. (a) Anteroposterior view showing a 1.7x1.4 cm nodular density at the level of aortic arch (red arrow). No other definitive nodules were seen. (b) Posteroanterior radiograph six months later, revealing the previously noted nodular density (red arrow) that remains unchanged and several new nodular opacities (white arrows) in the left upper and left lower lung fields.

**Figure 2:**
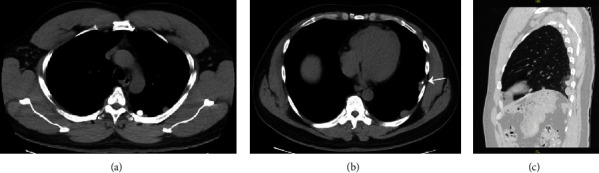
Computed tomography of the chest without contrast. (a) Axial view, mediastinal window at the level of the aortic arch reveals three pleural based nodules one of which is densely calcified, round, and smoothly marginated. (b) Axial view, mediastinal window of the lower thorax shows two pleural based smoothly marginated nodules of soft tissue density, one of which is partially calcified and lobulated (white arrow). (c) Sagittal view, lung window of left hemithorax showing four non-calcified pleural based nodules, one of which is lies along the left major fissure. Note the absence of the spleen.

A 49-year-old male presented for evaluation of lung nodules found incidentally on imaging. His medical history was significant for a gunshot wound, which he sustained 26 years earlier, to the left paraspinal muscle exiting the left upper quadrant of the abdomen. As a result of this trauma, he required a splenectomy, partial hepatectomy, left nephrectomy, and an appendectomy. The patient presented to the ER for chest pain felt to be musculoskeletal in origin. A chest X-ray revealed a nodule posterior to the aortic knob. He did not have further evaluation of the nodule at that time. 6-months later, he again presented to the ER with abdominal pain and chest discomfort described as burning in nature. No significant etiology of his symptoms was identified. A chest X-ray in the ER again revealed a nodule at the level of the aortic knob which had not changed since the prior exam, however, several new nodular opacities in the left upper and lower lung fields were now apparent (Figure 1 A & B). A chest computed tomography (CT) scan without contrast revealed numerous pleural-based, smoothly marginated nodules of soft tissue density in the left hemithorax. One of these nodules was round with smooth borders and densely calcified, and another nodule was lobulated and partially calcified (Figure 2 A-C). The patient was then referred to pulmonary clinic for further evaluation. He had no respiratory or constitutional symptoms. Additional history revealed the absence of prior malignancy, tuberculosis, or asbestos exposure. Exam revealed the sequelae of prior trauma and surgery with multiple thoracic and abdominal scars. The exam was otherwise unremarkable. The lung fields were clear and there was no peripheral lymphadenopathy.

**Figure 3:**
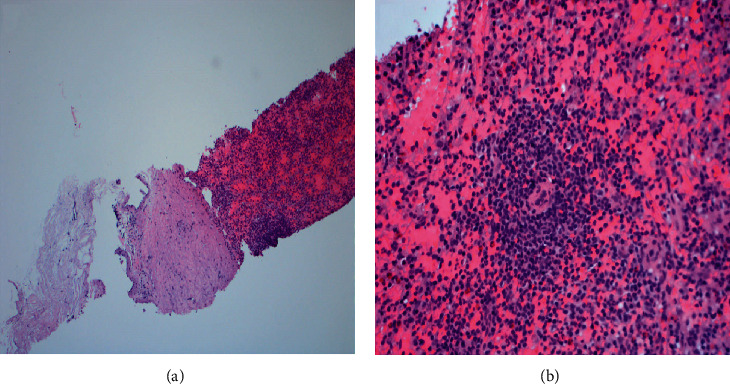
Pathology findings of the left pleural nodule showing (a) splenic tissue with a thick collagen band representing the splenic capsule (H&E stain, 100x). (b) Splenic tissue with congested blood-filled sinuses representing red pulp and lymphoid aggregates surrounding vascular structures representing white pulp (H&E stain, 400x).

Thoracic splenosis was considered, however, due to the atypical appearance of these lesions (as calcification was present) and the development of new nodules over a six month period, (despite the occurrence of splenic trauma 26 years prior), a transthoracic CT-guided biopsy was chosen over a nuclear scan. Sections of a pleural-based nodule showed splenic tissue with congested blood-filled sinuses representing red pulp and lymphoid aggregates surrounding vascular structures representing white pulp. No malignant cells or granulomas were identified and intrathoracic splenosis was diagnosed (Figure 3). Since the patient was asymptomatic, no intervention was felt necessary and the patient was followed for symptoms and growth. On a follow-up visit 6-months later, the patient remained asymptomatic and his chest X-ray remained unchanged.

## 3. Discussion

Thoracic splenosis is a rare entity. The lesions are frequently discovered incidentally on chest imaging and the patients are typically asymptomatic. Intrathoracic splenosis occurs as a result of a simultaneous rupture of the diaphragm and spleen. The splenic tissue is presumed to then cross into the left hemithorax and proliferate on the serous surface of the pleura and begin the seeding process [3]. The splenic implants are benign, round, smooth, or sessile pleural-based nodules commonly found in the left hemithorax [4]. On chest CT with and without IV contrast, the splenules will be similar in attenuation to the appearance of normal spleen. [5]. Microscopically, these nodules show lymphoid follicles with areas of red pulp and white pulp surrounded by a thick fibrous capsule [4, 6].

The diagnosis is challenging as the pleural-based nodules may be mistaken for an intrathoracic malignancy leading clinicians to perform invasive diagnostic procedures to obtain tissue such as a needle biopsy or a video-assisted thoracic surgery (VATS) biopsy [7]. The diagnosis can be made by technetium (Tc)-99 m colloid scintigraphy. The Tc-99 m sulfur colloid is administered intravenously and is taken up by the reticuloendothelial system. When used with single photon emission computed tomography (SPECT), an intense radionuclide uptake in areas of the scan is diagnostic for splenules [2, 8]. Other available nuclear medicine diagnostic tools include ferumoxides-enhanced magnetic resonance imaging which may provide higher spatial resolution while avoiding ionizing radiation [9]. In this case, given the atypical appearance of the CT and the evidence of progression over a six-month period of time, a biopsy was chosen over a nuclear scan.

Splenosis is a benign condition as it is generally slow-growing and non-invasive [10]. Nonetheless, complications can occur. If the splenic tissue is large enough, thoracic splenosis can present as a pulmonary space-occupying lesion that produces symptoms such as shortness of breath, hemoptysis, and chest pain [11–13].

Surgical resection of the splenic tissue is generally not recommended in asymptomatic patients. The protective role of splenic nodules and their ability to replace the removed spleen is controversial. The splenic tissue present in splenosis is active and receives blood supply from surrounding tissue [6]. Keeping the ectopic splenic tissue may offer a degree of immunologic defense, such as protection against post-splenectomy bacterial infections and a decrease in subsequent sepsis in these otherwise asplenic patients [14–16]. Auto-transplantation of splenic tissue into the greater omentum or liver has been performed at the time of surgery to try and preserve normal splenic function where splenectomy is unavoidable [17–20].

A review of the literature only reported two cases of calcification in intrathoracic splenosis, one which noted “stippled” calcification [21, 22]. Splenic calcification can be seen in the setting of splenic infarction, such as in sickle cell disease and cardiac thromboembolic disease [23, 24]. One can speculate that infrequently, splenosis may be compromised by inadequate vascular supply leading to infarction and necrosis.

## 4. Conclusions

Thoracic splenosis is the result of pleural seeding of splenic deposits, usually after thoracoabdominal trauma. Chest CT features are usually single or multiple non-calcified pleural nodules [25] and are often misinterpreted as malignancy on initial imaging. We present a patient with thoracic splenosis presenting with calcified and non-calcified pleural based nodules. To the best of our knowledge, only two cases of calcification in intrathoracic splenosis have been reported in the literature (one of which noted the presence of “stippled” calcification) [21, 22]. No prior images of calcification in intrathoracic splenosis have been published. The presence of calcification should not dissuade one from considering this diagnosis.

## Data Availability

All relevant data have been included in the manuscript. Further details and information about the case are available upon request.
